# “I WILL SURVIVE” A Construct Validation Study on the Measurement of Sustainable Employability Using Different Age Conceptualizations

**DOI:** 10.3389/fpsyg.2017.01690

**Published:** 2017-09-27

**Authors:** Pascale M. Le Blanc, Beatrice I. J. M. Van der Heijden, Tinka Van Vuuren

**Affiliations:** ^1^Human Performance Management Group, Department of Industrial Engineering, Eindhoven University of Technology, Eindhoven, Netherlands; ^2^Faculty of Psychology, Stockholm University, Stockholm, Sweden; ^3^Institute for Management Research, Radboud University, Nijmegen, Netherlands; ^4^Open University of the Netherlands, Heerlen, Netherlands; ^5^Kingston Business School, Kingston University, London, United Kingdom; ^6^Department of Organisation, Faculty of Management, Science and Technology, Open University of the Netherlands, Heerlen, Netherlands; ^7^APG Loyalis, Heerlen, Netherlands

**Keywords:** sustainable employability, calendar age, organizational age, functional age, life-span age, successful aging, ability, motivation and opportunity to continue working

## Abstract

Though the importance of sustainable employability throughout people's working life is undisputed, up till now only one attempt for a conceptual definition has been made (van der Klink et al., 2016). Following the suggestions to further refine and improve this definition recently put forward by Fleuren et al. (2016), we propose an approach to sustainable employability that is based on the Ability-Motivation-Opportunity (AMO) framework, and incorporates three indicators: the ability, the motivation, and the opportunity to continue working, respectively. As sustainable employability is considered to be an important aspect of successful aging at work, this study used four different conceptualizations of aging at work to set up convergent and divergent validity of our operationalization of sustainable employability: calendar age, organizational age (job and organizational tenure), functional age (work ability), and life-span age (partner and children). We formulated several hypotheses that were tested by analyzing data from an online survey among 180 employees from Dutch public service organizations who filled out a questionnaire on different age concepts, and their ability, motivation, and opportunity to continue working. Multiple regression analyses were performed, and results showed that the four conceptualizations of aging were differently related to the three indicators of sustainable employability. Life-span age, in terms of having children, had the strongest negative relationship with the ability to continue working, organizational age (i.e., organizational tenure) had the strongest negative relationship with the motivation to continue working, and functional age had the strongest negative relationship with the opportunity to continue working. Moreover, functional age was significantly negatively related to the other two indicators of sustainable employability too, while life-span age appeared to enhance the ability and motivation to continue working (in terms of having children) and the perceived opportunity to continue working (in terms of having a partner). Calendar age was only important for the opportunity to continue working and appeared to have a negative association with this outcome variable. These results lend support to our proposed operationalization of sustainable employability by showing that the three indicators are differently related to different age conceptualizations thus expanding previous research on the conceptualization of sustainable employability.

## Introduction

Given the aging and dejuvenization of the working population (Philips and Siu, 2012), and the expected shortages in employees' skills in the future (World Economic Forum, 2016), it is of utmost importance to focus on their employability and prolonging their working life until, or even beyond, their official retirement age. The use of the term “employability” goes back to the fifties of the previous century when it was supposed to be an important determinant for securing a job, in particular, to make sure that one had paid work in the (near) future (Feintuch, 1955). Over the past decades, researchers continuously adapted the conceptualization of employability to the existing labor market situation, and different purposes, interventions, target groups, measures, and activities were discerned (see Thijssen et al., 2008). The valuable historical outline drawn by Versloot et al. (1998) clarifies that there has been an evident shift over the years in what exactly the employer's stake might be. All in all, the notion of life-time employment has been gradually replaced by the notion of life-long employability (Hillage and Pollard, 1998; Forrier and Sels, 2003; Fugate et al., 2004; Rothwell and Arnold, 2007; Van der Heijden et al., 2009). In today's new economy, with its ever-increasing market pressures, leaner organizations, and rapid changes in requirements (Lazarova and Taylor, 2009), focusing upon sustainable employability or the sustainability of individuals' careers over time is of utmost importance (Van der Heijden and De Vos, 2015; Veld et al., 2015; Van Dam et al., 2017).

Employability research can be divided in input- and outcome-based approaches (see Forrier et al., 2015, for more detailed information). In this contribution, an outcome-based approach is used focusing on indicators of outcomes that are associated with the likelihood to continue working (see for instance Berntson et al., 2006; Rothwell and Arnold, 2007; Wittekind et al., 2010; De Cuyper et al., 2012; Vanhercke et al., 2014). As such, sustainable employability can be considered to be an important aspect of successful aging at work (Zacher, 2015), and denotes the degree to which an employee is willing and able to carry out his/her current and future work (Van Vuuren, 2011). Just like sustainable development is vital for retaining world's natural resources (United Nation's Brundtland Commission, WCED, 1987), sustainable employability is vital for retaining employees' (income) resources and their ability, motivation and opportunity to continue working. Individual workers' sustainable employability comprises that employees are working in such a way that they are able to meet their own needs and labor market requirements in the present, without compromising their ability to meet these in the future (see also Van der Heijden et al., 2016). It implies that workers are capable and motivated to handle changes over time, both in themselves and in the labor market (Sanders et al., 2015). In addition, according to van der Klink et al. (2016) sustainable employability means that workers enjoy the necessary conditions that allow them to make a valuable contribution to the organization as well as to their own benefit through their work, now and in the future, while safeguarding their health and welfare.

Recently, Fleuren et al. (2016) critically reflected on the latter definition of van der Klink et al. (2016) and give five suggestions to optimize this definition: (1) explain what aspects of employment constitute someone's sustainable employability as in its current definition by van der Klink et al. (2016), the concept seems to be predicted by itself in the form of a capability set; (2) do not deal with sustainable employability as a characteristic of both the person and the job, simultaneously, instead, one should disentangle them; (3) do not rely on the unpredicted assumption that achieving value in work inherently leads to sustainable employability; (4) do not formulate sustainable employability in such a way that the concept only applies to employed individuals; and (5) specify better how the inherently longitudinal dimensions of sustainable employability should be addressed.

Obviously, the sustainable employability concept is highly complex and one needs more than one indicator to adequately operationalize it. In particular, sustainable employability requires a work context that facilitates employees by providing opportunities to continue working and, at the same time, an employee's attitude and motivation to exploit these opportunities. Correspondingly, we argue that sustainable employability asks for three conditions to be met: first, employees need to have the *ability* to continue working; second, they need to have the *motivation* to continue working; and third, they need to have the *opportunity* to continue working (Semeijn et al., 2015; Van der Heijden et al., 2015). Ability, motivation and opportunity are the key components in the Ability-Motivation-Opportunity (AMO) framework of individual performance (Appelbaum et al., 2000; Boxall and Macky, 2009) which states that HRM practices should be designed in such a way that they stimulate all three components. Building upon the suggestions by Fleuren et al. (2016), we propose an approach to sustainable employability that is based on the AMO framework, thus adding to the scholarly literature in this field. We argue that sustainable employability is an individual attribute, which applies to all (potential) workers, including the unemployed and self-employed ones. In the current study, we will build upon the AMO distinction and will use three indicators. The first indicator refers to the degree to which individuals believe that they are able to work and to continue working, even beyond their retirement age (Van der Heijde and Van der Heijden, 2006; Geuskens et al., 2012). The second indicator refers to the motivation to work and to continue working (see also Geuskens et al., 2012). The third indicator comprises the opportunity to work now and in the future, or one's perceived labor market opportunities (Vanhercke et al., 2014, p. 593), and refers to “the individual's perception of his/her possibilities of obtaining and maintaining employment.” Thus, we propose that sustainable employability is an individual attribute, we do not use a definition in which antecedents are included, and we specify which aspects constitute someone's sustainable employability. We acknowledge that sustainable employability may have many causes, amongst others, whether people realize value in their work. Moreover, we argue that our definition of sustainable employability applies to all possible categories of workers, including the unemployed, and in our conceptualization the longitudinal dimension is incorporated as well by asking the employees about their expectations for the future.

In addition, we will investigate the relationships of four different age conceptualizations with the three distinct indicators of sustainable employability. Given the fact that earlier research on aging at work and sustainable employability is limited and conceptually diverse (see also Kooij et al., 2008), it is important to incorporate multiple subjective age conceptualizations, over and above calendar age, being an objective measure, only (see also Cleveland et al., 1997). Calendar age is just a proxy measure for many complex changes related to aging (Hall et al., 2007), and the older people get, the more experience they accumulate, and the more heterogeneous they become (Staudinger and Bowen, 2011). Subjective age or age perceptions reflect “characteristics of the individual (e.g., physical appearance), but they are also affected by the context in which a person works or interacts” (Cleveland et al., 1997, p. 240), and appear to be associated with individual characteristics, such as work competence, health and changes in major life roles (Cleveland and Shore, 1992). As such, these more specific aging measures might be a better predictor of (some of) the indicators of sustainable employability than calendar age.

To summarize, the main objective of our study is to investigate the association between aging and sustainable employability using a broader conceptualization for both constructs. More specifically, we will examine the convergent and divergent validity of our operationalization of sustainable employability by relating the three indicators of sustainable employability outlined above with four different age conceptualizations. In this way, we aim at a better understanding of the conceptualization and operationalization of sustainable employability. As will be explicated in more detail in the paragraphs below, we expect differential relationships of the four different conceptualizations of age with the three indicators of sustainable employability. Therefore, the three indicators of sustainable employability are not combined into one overall construct. Moreover, based on the results of our study, we discuss possible directions for management, HR practitioners and workers themselves on how to protect and enhance sustainable employability over the life span.

## Hypotheses

### Toward different conceptualizations of aging at work

*Aging at work* can be seen as a multi-dimensional process indicating changes in psychological, physical, social as well as societal functioning across time (De Lange et al., 2006; Kooij et al., 2008), and is argued to affect individual employees on the personal, organizational, and societal levels (cf. Sterns and Miklos, 1995; Kooij et al., 2008). Sterns and Doverspike (1989) proposed five different approaches comprising chronological, organizational, functional, psychosocial, and life-span development to measure age-related changes, due to health, career stage, and family status, among others, across time. As individuals with the same chronological (or objective) age may differ in terms of these age-related changes, a more elaborate conceptualization of aging, including subjective measures, is needed to better understand the impact of aging on sustainable employability.

*Calendar* age, or chronological age, refers to the time passed since one's date of birth. *Organizational* age refers to the aging of individuals in jobs and organizations, which is more commonly referred to as seniority, and job or organizational tenure (years of service), or as career stage (Kooij et al., 2008). *Functional* or performance-based age comprehends a worker's ability to perform certain tasks on a daily basis (Sharkey, 1987). *Psychosocial* or subjective age comprises the social perception of age, and refers to how old an individual feels, looks and acts, with which age cohort the individual identifies, and how old the person desires to be (Kaliterna et al., 2002; Stephan et al., 2012). It also involves age norms applied to an individual with respect to an occupation, company, or society (e.g., stereotypes of older workers). The concept of *life-span* age emphasizes the intra-individual changes due to individuals moving through (older) adulthood, and relates to behavioral changes at any point in the life cycle. Life-span age can, for example, be measured by life stage or family status (number and age of dependents, marital status; Sterns and Doverspike, 1989; Sterns and Miklos, 1995; De Lange et al., 2006). Substantial events—such as getting married, having children, and experiencing loss of a loved one all mark transitions of one position or “social identity” to another one.

### The impact of aging at work on sustainable employability

Over the past decades, *life-span theories* have started to focus on more complex, multi-dimensional or dynamic conceptualizations of the aging process (De Lange et al., 2015). Concrete, both the life-span theory of Selection Optimization and Compensation (Baltes, 1987; Baltes and Baltes, 1990; Baltes et al., 1999) and Socio-emotional Selectivity Theory (Carstensen, 2006) highlight the importance of conservation of resources through self-regulatory compensatory goal-related choices and coping strategies. However, despite recent developments in life-span theories incorporating broader age conceptualizations, an elaborate overview of earlier empirical research examining their relationships with sustainable employability is still missing. Therefore, we cannot borrow from a firm and sound theoretical framework to support our assumptions; yet, we will build upon all relevant empirical evidence that, to the best of our knowledge, exists. In the following sections, we will go into the scholarly literature and will formulate 12 hypotheses.

### Calendar age and sustainable employability

Calendar, or objective, age is by far the most widely used conceptualization to study the impact of aging on sustainable employability. The Netherlands Working Conditions Cohort Survey (Geuskens et al., 2012) investigated the differences between age groups of older workers regarding their ability and motivation to continue working. This survey is based on samples aged 45 and older, and showed a positive relationship between calendar age and perceived ability to continue working until the age of 65 (Ybema et al., 2010; Geuskens et al., 2012). Employees between 60 and 63 years more often indicated that they were able to continue working until the age of 65 than younger employees. According to Geuskens et al. (2012), this probably reflects a selection process, that is, the “healthy worker effect,” implying a selection of healthy older workers who remain in employment (McMichael, 1976). Oude Hengel et al. (2012) also found proof of this healthy worker effect in a large sample of Dutch construction workers between 15 and 64 years in the Netherlands Working Conditions Survey, where the older ones indicated to be more able to continue working in their current profession than the younger ones. As our current study sample consists of people who are currently (still) working, we hypothesize:

Hypothesis 1a: Calendar age is positively related to the ability to continue working.

Motivation to continue working is a relatively new concept (e.g., Kooij et al., 2014; Akkermans et al., 2016), and refers to both intrinsic and extrinsic motivation to work, and work values and their fulfillment (De Wind et al., 2015). Up to now, the concept has gained increasing attention given its importance for successful retention of older workers (Kanfer and Ackerman, 2004; Stamov-Roßnagel and Hertel, 2010; Kanfer et al., 2013), and, herewith as an indicator of the sustainable employability of the workforce.

Results of studies on the relationship between calendar age and the motivation to continue working are inconsistent. Geuskens et al. (2012) found that older age was positively related to the willingness to continue working until the age of 65 in a representative sample of the Dutch working population aged 45 and above. However, Oude Hengel et al. (2012) revealed that older construction workers were less willing to continue working than their younger colleagues. This latter finding is in line with the Socio-emotional Selectivity Theory (Carstensen, 1995), which states that individuals select and pursue goals in alignment with their (working) life's time horizon. In particular, in case individual workers have a limited future time perspective, they are inclined to seek psychological well-being and short-term benefits. On the contrary, when they view their remaining time as open-ended, individual goals to acquire knowledge, experience novelty, etc. become more important (Carstensen, 2006). Building upon Socio-emotional Selectivity Theory, we assume that older people perceive their remaining time and opportunities as more limited, and, as a result, are less motivated to continue working (see also De Lange et al., 2011). Specifically, we expect them to focus on different goals outside working life, such as deepening existing relations with people in their private life.

In a similar vein, the Selection Optimization and Compensation theory (Baltes and Baltes, 1990) proposes that, with advancing age, individuals will allocate fewer resources to growth, due to age-related losses in resources, such as the perception of time (e.g., Freund and Ebner, 2005). When people are younger, and time is perceived as expansive, open-ended, development goals aimed at optimizing the future are relatively more important (Bal et al., 2010). Reversely, in case of a limited future time perspective, the utility of further development is likely to decline, as individuals perceive that development goals are unlikely to be attainable in the limited lifetime remaining. Otherwise stated, age-related decline in future time perspective is claimed to shift attention away from development goals, and, as a consequence, reduce the strength of growth-related motives at work and self-regulation strategies (De Lange et al., 2011; Kanfer et al., 2013), herewith reducing the employee's motivation to continue working. Based on this line of reasoning, we hypothesize the following:

Hypothesis 1b: Calendar age is negatively related to the motivation to continue working.

Self-perceived labor market opportunities or perceived employability (Van der Heijden et al., 2009; Forrier et al., 2015) lie at the core of a positive process that leads to optimal employee functioning (Vanhercke et al., 2014). Previous studies, in general, have found negative relationships between calendar age and perceived labor market opportunities (e.g., Van der Heijden et al., 2009; De Cuyper et al., 2011; Van Vuuren et al., 2011). The lower employability of older workers can be due to age discrimination in organizations as shown in fewer investments in older employees, less appreciation of older employees and less opportunities to engage in interesting tasks, job transitions and development activities (Finkelstein and Farrell, 2007; Billet et al., 2011; Truxillo et al., 2012). Therefore, we hypothesize the following:

Hypothesis 1c: Calendar age is negatively related to the opportunity to continue working.

### Organizational age and sustainable employability

In line with what has been stated in the previous paragraphs regarding the healthy worker effect (McMichael, 1976), we hypothesize the following:

Hypothesis 2a: Organizational age is positively related to the ability to continue working.

Although Kooij et al. (2008), on the basis of a meta-analysis of 24 empirical and nine conceptual studies, concluded that most age-related factors can have a negative impact on the motivation to continue to work of older people, they were less certain about the impact of organizational age. On the one hand, they found that organizational aging has a negative effect on the motivation to continue working because of skill obsolescence, but, on the other hand, a positive effect as well, probably due to the rise in salary with increasing seniority. Given these mixed findings, we have formulated the following non-directional hypothesis:

Hypothesis 2b: Organizational age is associated with the motivation to continue working.

Regarding job and organizational tenure, previous research has shown the danger of experience concentration (Thijssen, 1996) or skill obsolescence (Schalk et al., 2010) which comprises that people have been specializing themselves so strongly, and over such a long period of time that it is hard for them to find or to learn another job and to stay employable (see also Van der Heijden and Thijssen, 2003). As the labor market has changed tremendously over the past years, due to a combination of the ever-increasing speed in developments, increasing globalization and demands on productivity, creativity, and flexibility, employees are required to continuously update their occupational knowledge and skills (Berntson et al., 2006; Van der Heijde and Van der Heijden, 2014) during their entire career. The increasingly volatile “new” work environment (Berntson et al., 2006) requires that, in order to remain competitive on the internal and external labor market (Klein Hesselink and Van Vuuren, 1999), employees need to regularly change tasks, jobs, and/or organizations. Therefore, we hypothesize:

Hypothesis 2c: Organizational age is negatively related to the opportunity to continue working.

### Functional age and sustainable employability

Functional age has to do with employees' ability to perform certain tasks on a daily basis (Sharkey, 1987), and reflects cognitive abilities and physical health (Sterns and Doverspike, 1989; Kooij et al., 2008). Geuskens et al. (2012) found that poor physical health (i.e., the occurrence of musculoskeletal symptoms) is negatively related to the ability to continue working. In an 11-year follow-up study among 818 active employees, Ilmarinen et al. (1997) found that the mean Work Ability Index (WAI) declined significantly for both genders due to chronological aging and work context factors. As the normative age trajectory is a decline in health and work ability, we hypothesize the following:

Hypothesis 3a: Functional age is negatively related to the ability to continue working.

Functional age also has a negative impact on the motivation to continue working (Kooij et al., 2008). More specifically, studies by Oude Hengel et al. (2012) and by Geuskens et al. (2012) revealed that poor health was negatively related to the motivation to continue working until the age of 65 among respectively construction workers and among older employees in general. Possibly, this can be explained by the fact that employees with poor health have to invest more effort in performing their work, which is depleting not only their physical but also their motivational resources. As the normative age trajectory is a decline in health and work ability, we hypothesize:

Hypothesis 3b: Functional age is negatively related to the motivation to continue working.

Finally, work ability appeared to have a positive relationship with employability in Dutch studies among employees in primary education (Van Vuuren et al., 2011; Van Vuuren and Marcelissen, 2013). As the normative age trajectory is a decline in health and work ability, and people with a decline in work ability will generally be less likely to keep their current job or find a new one, we hypothesize:

Hypothesis 3c: Functional age is negatively related to the opportunity to continue working.

### Life-span age and sustainable employability

Life-span age stands for the sequence of positions that a person holds over a period of time (Kanfer et al., 2013), and has been measured in very different ways: for instance, as having a partner or not, having a partner with a paid job or not, or having children (at home or not. Moreover, as already mentioned before, the older people get, the more experience they accumulate, and the more heterogeneous they become (Staudinger and Bowen, 2011). Given the pluriformity in private life situations, it is difficult to translate the findings of these studies into directional hypotheses. In the present study, we decided to include two of the most significant aspects of people's private lives: having a partner or not, and having children (at home) or not. Firstly, marital status and living arrangements, along with changes in these in mid-life and older ages, have implications for an individual's health and mortality. Literature on health and mortality by marital status has consistently identified that unmarried individuals generally report poorer health and have a higher mortality risk than their married counterparts, with men being particularly affected in this respect (Robards et al., 2012). Whereas marriage appears good for everyone's health and well-being, a review of Umberson et al. (2010) concludes that parenthood has significant effects on well-being over the life course, although for some there are positive and for others—such as women, unmarried parents, and individuals with lower social economic status—negative effects. Life-span age also matters as the ability to (not) continue working is related to the financial possibility to retire early (Proper et al., 2009; De Wind et al., 2014). Having to carry the financial burden of paying maintenance for their spouse or paying their children's college tuition may reduce the ability of partners and parents to retire early. On the other hand, the social support from a partner can enhance the perceived ability to continue working (De Wind et al., 2015). As explained above, we decided to refrain from formulating directional hypotheses for life span age. Based on the outcomes of the above studies, we hypothesize:

Hypothesis 4a: Life-span age is associated with the ability to continue working.

Kooij et al. (2008) concluded that one's partner's wishes and increased value placed on leisure time reduced workers' motivation to continue working and encouraged their decision to retire. Similarly, Van Dam et al. (2009) showed that employees who felt a pressure from their spouse to retire early had a strong intention to leave the work force before the official retirement age. De Wind et al. (2015) found that a positive attitude of the partner with respect to early retirement, and not having a partner, were associated with early retirement. Geuskens et al. (2012) found that employees having a partner without a paid job were more often willing to continue working until the age of 65.

Hypothesis 4b: Life-span age is associated with the motivation to continue working.

The perceived opportunity to continue working is also related to life-span age. McQuaid and Lindsay (2005) reported that individual household circumstances, such has having direct caring responsibilities for children, and financial, emotional, and/or time commitments to family members may affect the employability of people and thus their opportunity to continue working.

Hypothesis 4c: Life-span age is associated with the opportunity to continue working.

## Methodology

### Participants and procedure

Data were collected by means of an e-questionnaire that was distributed among employees of Dutch public service organizations that are responsible for water management in terms of consultation, facilitation, technical execution, and management of water protection. Their jobs are very diverse, ranging from muskrat catcher, inspector of waterworks, receptionist, human resource consultant, crisis coordinator, purification technician, environmental inspector, water level inspector to chairman of the water board. On average, their job demands can be classified as low physical demands, moderate emotional demands and high cognitive demands. Confidentiality and anonymity of responses were guaranteed. Questionnaires were distributed in one public service organization among 140 employees of which 118 employees responded (response rate of 84%). In addition, about one third of the study sample (*n* = 62) replied individually to a call on the website of the labor market and training fund of the Dutch water boards. The final sample was made up of 120 male (67%) and 60 female workers (33%), 41.2% of the participants worked full-time.

### Measures

#### Age operationalizations

*Calendar age* (objective age) was measured by asking the person for his/her date of birth, and ranged from 26 to 64 years. The mean calendar age was 48.99 years (*SD* = 8.42).

*Organizational age* was assessed by means of two separate items: “Since when are you working in your current organization?” and “Since when are you working in your current job?” Respondents' organizational tenure ranged from 1 to 42 years, whereas their job tenure ranged from 1 to 38 years. On average, they were working 16.54 years (*SD* = 9.98) in their current organization, and 11.46 years (*SD* = 8.56) in their current job.

*Functional age* was operationalized in terms of work ability, which was measured by means of the WAI that has been proven to be a good predictor of one's work ability (in the future) (Tuomi et al., 1994). The WAI is based on a series of questions that takes into consideration both the physical and mental demands of work and the health and resources of the employee (Ilmarinen et al., 2005) and consists of seven items that are scored in the following way: (1) one's current work ability compared with one's life-time best (scored on a 0–10 points' rating scale); (2) work ability in relation to the demands of the job (scored on a 2–10 points' rating scale); (3) number of current diseases diagnosed by a physician (scored on a 1–7 points' rating scale); (4) estimated work impairment due to diseases (scored on a 1–6 points' rating scale); (5) sick leave during the past year (scored on a 1–5 points' rating scale); (6) one's own prognosis of work ability 2 years from now (scored with 1, 4, or 7 points); and (7) mental resources (scored on a 1–4 points' rating scale). The WAI is calculated by summing the points of the seven items (possible score ranging from 7 to 49 points), and can be divided into the following four classes: poor outcome (7–27 points), moderate outcome (28–36 points), good outcome (37–43 points), and excellent outcome (44–49 points) (Tuomi et al., 1994). In earlier research, scholars found a Cronbach's α of 0.70 (Alavinia, 2008), and De Zwart et al. (2002) found that the test-retest reliability of the WAI was acceptable. In the current study, the Cronbach's α was 0.75. The respondents' WAI scores ranged from 26 to 49 with an average WAI of 39.93 (*SD* = 5.05). As the normative age trajectory is decline in health and work ability, we reversed the WAI-scores for further analyses.

*Life-span age* was measured with one item: “What is your private life situation?” and was scored using the following answering categories: (a) married or cohabiting without children; (b) married or cohabiting with children living at home; (c) married or cohabiting with children living elsewhere; (d) single without children; (e) single parent with children living at home; (f) single parent with children living elsewhere. Based on the respondents' answers, we composed three variables: partner (0 = single; 1 = married or cohabiting), and children (0 = no; 1 = yes) and children at home (0 = no; 1 = yes). Eighty-five percent of the respondents (*N* = 153) were living with a partner, 10.6% (*N* = 19) did not have a partner, and for 4% (*N* = 8) their marital status was unknown. With respect to children, 80% (*N* = 144) of the respondents did have children. In addition, 57% (*N* = 103) had children living at home. For 4% of respondents (*N* = 8), parental status was unknown.

#### Sustainable employability

*Ability to continue working* was assessed by means of one item (Van den Bossche et al., 2008; Koppes et al., 2011; Geuskens et al., 2012; Ybema et al., 2014): “Until what age do you consider yourself—physically and mentally—able to continue your current work/job?” This question (as well as the one below about the motivation to continue working) has been used for years already in large-scale TNO studies, i.e., NWCS (Netherlands Working Conditions Survey; Koppes et al., 2011), and STREAM (Studies on Transitions in Employability, Ability and Motivation; Ybema et al., 2014). The NWCS is a large-scale periodical investigation into the working conditions of Dutch employees. Ten surveys have been performed to date, in 2003 and 2005–2016. Some 23,000–38,000 employees per year have responded to the surveys (Van den Bossche et al., 2008; Hooftman et al., 2016). Respondents were assigned a report grade based on their response (1 < 60 years and 10 > 66). A 10 was assigned to a response of 66 years and above because the retirement age in the Netherlands is to be increased gradually and will reach 67 years and 3 months in 2022. After 2022, the retirement age will be linked to the average life expectancy.

*Motivation to continue working* was assessed by means of one item (Van den Bossche et al., 2008; Koppes et al., 2011; Geuskens et al., 2012; Ybema et al., 2014): “Until what age do you want to keep on working?” Respondents were again assigned a report grade based on their response (ranging from 1 = age < 60 years to 10 = 66 years or more).

*Opportunity to continue working* was assessed by means of a three-item scale on perceived internal and external employability by Verboon et al. (1999) and Veld et al. (2015) that were all scored on a five-point Likert scale ranging from: (1) absolutely not to (5) absolutely. An example item for external employability is: “I am confident that it is easy for me to find an attractive new job in a different organization.” Cronbach's α was 0.79.

### Analyses

First, we computed bivariate correlations between the different age operationalizations, to explore how they were interrelated. Next, we studied the correlations between the different age conceptualizations and the indicators of sustainable employability. Subsequently, we determined the relative strength of each of these relationships by performing multiple regression analyses, for each of the three indicators of sustainable employability separately, with the different age conceptualizations as predictors.

## Results

### Correlations

The results for the bivariate correlations between all study variables are presented in Table 1. From Table 1, it becomes clear that calendar age was significantly positively related to both aspects of organizational age, i.e., organizational tenure (*r* = 0.53; *p* < 0.01) and job tenure (*r* = 0.33; *p* < 0.01), and significantly positively related to functional age, i.e., work ability (*r* = 0.19; *p* < 0.05). Logically, the two aspects of organizational age were positively related to one another (*r* = 0.35; *p* < 0.01). Of the three aspects of life-span age, having children was positively related to calendar age (*r* = 0.17; *p* < 0.05), whereas having children at home was negatively related to calendar age (*r* = −0.24; *p* < 0.01). Having children was significantly positively related to having a partner (*r* = 0.17; *p* < 0.05). Finally, having children and having children at home were significantly positively interrelated (*r* = 0.56; *p* < 0.01). The ability to continue working was significantly positively related to the motivation to continue working (*r* = 0.47; *p* < 0.01), whereas the motivation to continue working and the opportunity to continue working were significantly positively related too (*r* = 0.21; *p* < 0.01).

**Table 1:** Correlations between the study variables.

**Variables**	***Mean***	***SD***	**1**	**2**	**3**	**4**	**5**	**6**	**7**	**8**	**9**	**10**
1. Calendar age	48.9	8.41	–									
2. Job tenure	11.46	8.56	0.33^**^	–								
3. Organizational tenure	16.53	9.98	0.53^**^	0.35^**^	–							
4. Functional age^4^	39.93	5.05	0.19^*^	0.09	−0.03	–						
5. Partner^1^	–	–	0.06	−0.06	−0.06	0.09	–					
6. Children ^2^	–	–	0.17^*^	0.00	0.11	0.00	0.17^*^	–				
7. Children at home^3^	–	–	−0.24^*^	0.02	−0.11	−0.06	0.13	0.56^**^	–			
8. Ability cont. working	8.09	2.78	0.01	−0.13	−0.13	−0.25^**^	0.01	0.17^*^	0.02	–		
9. Motivation cont. working	8.47	2.59	−0.19^*^	−0.09	−0.29^**^	−0.17^*^	0.12	0.11	0.09	0.47^**^	–	
10. Opportunity cont. working	3.78	2.04	−0.32^**^	−0.07	−0.16^*^	−0.29^**^	0.07	−0.10	0.12	0.10	0.21^**^	–

1 0 = no partner, 1 = partner;

2 0 = no children, 1 = children;

3 0 = no children at home, 1 = children at home;

4 measured as WAI-reversed;

* *p < 0.05*,

** *p < 0.01, two-tailed; N = 180*.

*Calendar age* was significantly negatively related to the motivation (*r* = −0.19; *p* < 0.05) as well as to the opportunity to continue working (*r* = −0.32; *p* < 0.01). Regarding the two aspects of *organizational age*, results showed that organizational tenure was significantly negatively related to both the motivation (*r* = −0.29; *p* < 0.01) and the opportunity to continue working (*r* = −0.16; *p* < 0.05). Job tenure, on the other hand, was not significantly related to any of the three indicators of sustainable employability. *Functional age* was significantly negatively related to all three indicators of sustainable employability, i.e., ability to continue working (*r* = −0.25; *p* < 0.01), motivation to continue working (*r* = −0.17; *p* < 0.05), and opportunity to continue working (*r* = −0.29; *p* < 0.01). Finally, as regards *life-span age*, only having children was significantly positively related to the ability to continue working (*r* = 0.16; *p* < 0.05).

### Multiple regression analyses

In Table 2, the outcomes of the regression analyses are presented.

**Table 2:** Multiple regression analysis of the different age conceptualizations on the three indicators of sustainable employability.

	**Ability to cont. working**		**Motivation to cont. working**		**Opportunity to cont. working**	
	***R*^2^**	**β**	***R*^2^**	**β**	***R*^2^**	**β**
	0.17		0.19		0.20	
Calendar age		0.13		−0.04		−0.26^*^
Organizational tenure		−0.25^**^		−0.35^**^		−0.03
Job tenure		−0.07		0.03		0.06
Functional age^4^		−0.28^**^		−0.20^*^		−0.27^**^
Partner^1^		0.00		0.13		0.18^*^
Children^2^		0.29^**^		0.21^*^		−0.09
Children at home^3^		−0.15		−0.09		0.04
Model F		F_(7, 151)_ = 4.43^**^		F_(7, 151)_ = 5.02^**^		F_(7, 151)_ = 5.51^**^

1 0 = no partner, 1 = partner;

2 0 = no children, 1 = children;

3 0 = no children at home, 1 = children at home;

4 measured as WAI-reversed;

* *p < 0.05*,

** *p < 0.01; N = 180*.

The *ability to continue working* was significantly positively related to having children (β = 0.29; *p* < 0.01), and significantly negatively to functional age (β = −0.28; *p* < 0.01) and organizational tenure (β = −0.25; *p* < 0.01). The *motivation to continue working* was significantly negatively related to organizational tenure (β = −0.35; *p* < 0.01), and to functional age (β = −0.20; *p* < 0.01), whereas it was significantly positively related to having children (β = 0.21; *p* < 0.05). Finally, *the opportunity to continue working* was significantly negatively related to calendar age (β = −0.26; *p* < 0.01) and to functional age (β = −0.27; *p* < 0.01), whereas it was significantly positively related to having a partner (β = 0.18; *p* < 0.05).

## Discussion

In most Western countries official retirement ages are rising, therefore protecting and enhancing workers' sustainable employability has become a top priority. However, up till now, only one profound attempt to define sustainable employability conceptually has been made (van der Klink et al., 2016). In the current paper, we propose an approach to sustainable employability that is based on the AMO framework of individual performance, and incorporate three indicators: the ability, the motivation, and the opportunity to continue working, respectively. As the three indicators turned out to be related but not too highly, it supports our argument that these three indicators measure different facets of the overall construct of sustainable employability. As sustainable employability can be considered to be an important aspect of successful aging at work, this study used four different conceptualizations of aging at work to set up convergent and divergent validity of our operationalization of sustainable employability: calendar age, organizational age (job and organizational tenure), functional age (work ability), and life-span age (partner and children). Our results showed that the distinguished age conceptualizations were indeed differently related to the indicators of sustainable employability. Life-span age, in terms of having children, had the strongest negative relationship with the ability to continue working, organizational age (i.e., organizational tenure) had the strongest negative relationship with the motivation to continue working, and functional age had the strongest negative relationship with the opportunity to continue working. Moreover, functional age was significantly negatively related to the other two indicators of sustainable employability too, while life-span age appeared to enhance the ability and motivation to continue working (in terms of having children) and the perceived opportunity to continue working (in terms of having a partner). Calendar age was only significantly related to the opportunity to continue working and appeared to have a negative association with this outcome variable. Thus, our results lend support to our proposed operationalization of sustainable employability and add to the debate on the definition of this concept. Secondly, most scientific research on aging at work has exclusively focused on the impact of calendar or objective age on employee well-being and functioning. By considering several alternative age conceptualizations, we were better able to capture the full complexity of the aging process. In this way, our study expands previous research on different conceptualizations of employee age in an organizational context, and their implications for successful aging on the job. Thirdly, our study helps to understand the construct of sustainable employability better as it provides a theoretical framework (the AMO model) to research it and to empirically test relationships with other constructs in the suggested nomological network of sustainable employability.

With respect to calendar age, only one out of the three hypotheses was confirmed. Calendar age was negatively related to the perceived opportunity to continue working. Whereas we found a negative bivariate correlation between calendar age and the motivation to continue working, interestingly, this relationship turned out to be non-significant after controlling for the three other age conceptualizations in the regression analysis. A possible explanation might be a so-called spurious correlation (Simon, 1954) between objective age and motivation to continue working caused by confounding effects of the subjective operationalizations of age. Earlier research found inconsistent results too. Geuskens et al. (2012) explained the positive relationship between calendar age and the motivation to continue working by means of the so-called healthy worker effect. The findings of Oude Hengel et al. (2012), who found a negative relationship, may be explained by the Socio-emotional Selectivity Theory, indicating that older workers are inclined to focus on different goals, outside working life. Obviously, longitudinal work is necessary to better understand the underlying mechanisms and possible processes linking calendar age and motivation to work.

Concerning organizational age, only one out of our three hypotheses was confirmed. Contrary to our expectation, organizational age, measured as organizational tenure, was negatively instead of positively related to the ability to continue working. The phenomenon of “experience concentration” (Thijssen, 1996) or skill obsolescence (Schalk et al., 2010) might be an important reason for this. The qualifications that are required for a job have become more and more complex and keeping abreast of new developments is needed in present-day working life. If employees do not succeed in updating their skills and knowledge during their entire career, their ability to continue working will be reduced. In addition, the reduction in early retirement possibilities may also provide less room for the healthy worker effect. Moreover, we found that organizational age was negatively related to the motivation to continue working. This may also be ascribed to the harmful effect of skill obsolescence, which apparently is more impactful on the worker's willingness to go on in comparison with positive effects such as the rise in salary with increasing seniority. In addition, as sustainable employability is a broad construct, involving work motivation, the negative relationship could also be a reflection of less motivated, or achievement oriented employees to not look for new opportunities. Contrary to our hypothesis, organizational age did not explain a significant amount of variance in perceived opportunity to continue working. Several factors might moderate this relationship, for example, aspects of work self-efficacy (Stajkovic and Luthans, 1998), perceived external employment opportunities (Rothwell and Arnold, 2007), and different types of organizational commitment (affective, normative, and continuance, see Meyer and Allen, 1991).

All three hypotheses for functional age were confirmed. In line with earlier research, we found that functional age decreases the ability, motivation and opportunity to continue to work (Sterns and Doverspike, 1989; Kooij et al., 2008; Geuskens et al., 2012). Given the cross-sectional approach of our study, further research using longitudinal designs is needed in order to shed more light on the mechanisms underlying this relationship. As sustainable employability is a rather broad construct, based on this empirical work we cannot exclude possible effects of underlying constructs such a general health, and energy levels, to mention but a few.

Concerning life-span age, we could partly confirm all three hypotheses. Having a partner, goes together with the self-perceived opportunity to continue working (see also McQuaid and Lindsay, 2005). Having children on the other hand, appeared to be positively related to both the ability and to the motivation to continue working. Earlier studies showed mixed results regarding the relationship between life-span age and the ability and motivation to continue working which may be caused by the large variety in operationalizatons of life-span age that have been used in different studies (Proper et al., 2009; De Wind et al., 2014, 2015). As already mentioned in the section on Hypotheses, research on the relationship between life-span age and health and mortality finds mixed results too. Marriage appears to be good for everybody, but parenthood does not have positive effects for all. Another possibility is that, given the fact that, nowadays, traditional life trajectories are less prevalent in comparison with the past, especially when the life-span increases, substantial events, such as marriage and having children might be less reliable markers of life-span with age. Obviously, more in-depth scholarly work taking into account the effect of all kinds of family constellations, incorporating financial, emotional, and time demands, is needed to better understand the impact of life-span age.

The present study has some limitations. Firstly, all data have been collected using a quantitative self-rating approach opening up the possibility of response set consistencies. Notwithstanding this limitation, we believe that using age measures based on the perceptions of employees themselves over and above calendar age has increased our insights on the possible role of age. In addition, by using self-perceived employability, which forms the basis of a mechanism leading to enhanced performance over the life-span (Vanhercke et al., 2014), our approach has high value in the light of a better understanding of sustainable careers (Van der Heijden and De Vos, 2015). Still, future studies might apply qualitative work as well, and include other-ratings of some study variables (e.g., 360° feedback or assessments by others for the measurement of the three indicators of sustainable employability). In addition, as both ability and motivation to continue working have been operationalized by one-item measures (a practice that should not be unequivocally rejected; see Wanous and Reichers, 1996), being a less time-consuming effort which was assumed to have more face validity in this case (see Nagy, 2002), future work using multi-item scales is called for in order to enable us to compare psychometric qualities.

Secondly, research using multi-wave designs can provide more specific information about the stability and change of the variables, and about cross-lagged (i.e., over time) relationships compared with our cross-sectional approach (Spurk and Abele, 2014). Related to this, reciprocal relationships between age conceptualizations and indicators for sustainable employability might be taken into account too. For example, it might be that more employable individuals are also more actively searching for opportunities to protect their functional age and or to adjust their private life situation to their career ambitions, and therefore increase their ability, motivation or opportunity to continue working. As a result, they might even become more employable over time, what would result in positive reciprocal relations between the study variables.

Thirdly, data have been collected among employees holding a variety of jobs but within one specific sector, and therefore future research among different occupational groups should be performed in order to test the generalizability of our results. For instance, public service employees may have a lesser need for employability due to more job security and better unemployment benefits than employees in the private sector. Moreover, the respondents in our sample had relatively high organizational and job tenure, so future research should use samples that are more diverse in this respect.

Finally, more scholarly work is needed to investigate the impact of psychosocial or subjective age. For instance, empirical research could focus on the impact of age stereotypes on discriminatory cultures in working organizations, and their effects on sustainable employability. Another promising path of scholarly work in this field concerns socially generated age effects, resulting from age norms (see for instance Lawrence, 1988) and relational demography (Shore et al., 2003). Also, we should investigate the extent to which national culture influences psychosocial or subjective age, and age norms. Finally, responding to the complexity of age and generations, more research is needed in order to better understand how, to what extent and under which circumstances age management policies might be effective in the light of managing age diversity and sustainable careers (Pitt-Catsouphes et al., 2011; Segers et al., 2014).

It goes without saying that chronological or objective age is something one cannot change. However, both individuals and organizational stakeholders (direct supervisor, management, and HR representatives) can intervene in the employee's organizational, functional and, to a lesser extent, life-span age to protect and enhance his/her sustainable employability. Sound interventions at the workplace focusing on lifestyle, health and work ability are advised to protect one's ability, motivation and opportunity to work. Also, we suggest measures to enhance the employee's mobility and career development aimed at reducing the possibly negative effects of organizational age, due to experience concentration. Only in case all parties involved do their utmost to promote workers' sustainable employability, their chances to survive in the current-day dynamic working environment are optimized. Hopefully, our attempt to conceptualize sustainable employability, and to substantiate our conceptualization by demonstrating different associations with age conceptualizations, stimulates future research in the field of both (the management of) employability and aging at work.

## Author contributions

All three authors (PL, BV, and TV) contributed equally to the drafting of the manuscript (research question, propositions) and to the writing of the manuscript. Data were collected by author TV. Data analysis was performed by author PL.

### Conflict of interest statement

The authors declare that the research was conducted in the absence of any commercial or financial relationships that could be construed as a potential conflict of interest.
